# Accelerated biological aging and its hallmarks in DNA methylation drive the association between unhealthy lifestyles and the onset of colorectal cancer

**DOI:** 10.1016/j.ebiom.2025.106005

**Published:** 2025-11-05

**Authors:** Jing Sun, Min Liu, Xiaoqian Zhang, Xinxuan Li, Jingyu Ye, Jianhui Zhao, Siyun Zhou, Haosen Ji, Yuqian Tan, Zilong Bian, Dongfeng Zhang, Malcolm G. Dunlop, Mingyang Song, Stephanie A. Smith-Warner, Hao Wu, Evropi Theodoratou, Qian Cao, Xue Li

**Affiliations:** a Department of Gastroenterology, Sir Run Run Shaw Hospital, Zhejiang University School of Medicine, Zhejiang, China; b Center of Clinical Big Data and Analytics of The Second Affiliated Hospital, School of Public Health, Zhejiang University School of Medicine, Hangzhou, Zhejiang, China; c Departments of Nutrition and Epidemiology, Harvard T.H. Chan School of Public Health, Boston, MA, USA; d Department of Colorectal Surgery, National Cancer Center/National Clinical Research Center for Cancer/Cancer Hospital, Chinese Academy of Medical Sciences and Peking Union Medical College, No. 17 Panjiayuan Nanli, Chaoyang District, Beijing, 100021, China; e Department of Epidemiology and Health Statistics, The School of Public Health of Qingdao University, Qingdao, Shandong Province, China; f Cancer Research UK Edinburgh Centre, Medical Research Council Institute of Genetics and Cancer, University of Edinburgh, Edinburgh, UK; g Colon Cancer Genetics Group, Institute of Genetics and Cancer, University of Edinburgh, Edinburgh, UK; h Division of Gastroenterology, Massachusetts General Hospital and Harvard Medical School, Boston, MA, USA; i Clinical and Translational Epidemiology Unit, Massachusetts General Hospital and Harvard Medical School, Boston, MA, USA; j Broad Institute of MIT and Harvard, Cambridge, MA, USA; k Department of Gastroenterology, National Clinical Research Center for Digestive Diseases, Changhai Hospital, National Key Laboratory of Immunity and Inflammation, Naval Medical University, Shanghai, China; l Centre for Global Health, Usher Institute, University of Edinburgh, Edinburgh, UK

**Keywords:** Biological aging, Unhealthy lifestyles, Colorectal cancer, DNA methylation, Mediation effect

## Abstract

**Background:**

Biological aging is thought to be associated with colorectal cancer (CRC), however, the mechanisms underlying are not fully understood. This study aimed to elucidate how biological aging contributes to the onset of CRC.

**Methods:**

We first performed a longitudinal cohort study (5448 incident CRC and 317,192 controls) to evaluate the relationships between biological aging (i.e., leukocyte telomere length, PhenoAge, Klemera-Doubal, homeostatic dysregulation [HD] score, frailty) and CRC, and assessed how it contributes to the association of modifiable risk factors with CRC using Cox regression models. Then, we performed Mendelian randomization (MR) studies to evaluate the relationship of biological aging with CRC risk from the epigenetic perspective (epigenetic aging clocks). Finally, a three-step MR analysis between aging-related DNA methylation (DNAm), gene expression, and CRC followed by colocalization analysis was performed to elucidate the CpGs/genes and possible pathways underlying aging and CRC.

**Findings:**

In the longitudinal cohort study, we found PhenoAge acceleration and HD score associated with increased CRC risk, and these associations were stronger for early-onset CRC (HR [95% CI]: 1.29 [1.07–1.55] for PhenoAge acceleration and 1.35 [1.08–1.69] for HD score) than late-onset CRC (HR [95% CI]: 1.06 [1.03–1.09] for PhenoAge acceleration and 1.05 [1.02–1.08] for HD score) (*P*_heterogeneity_<0.05). Accelerated biological aging partly mediated the adverse effect of unhealthy lifestyle and its components on CRC, with proportions of mediation ranging from 0.18% to 27.00%. In the epigenetic MR, genetically determined DNAm GrimAge was positively associated with CRC risk. Altered methylation at 15 aging-related CpGs was associated with CRC and was prioritized with high colocalization evidence. Four mapped genes (*TNF, BICC1, NCF2, DIP2B*) were significantly associated with CRC. The lower expression of *TNF*, *NCF2*, and *DIP2B* mediated the adverse effect of the methylation at cg04425624 and cg03037030 (*TNF*), cg09076123 (*NCF2*), and cg05512157 (*DIP2B*) on CRC, respectively, and higher expression of *BICC1* mediated the adverse effect of the methylation at 4 CpGs (cg08353444, cg23963517, cg06424110, cg09578524) on CRC.

**Interpretation:**

This study found that accelerated biological aging was associated with a higher risk of CRC and implied potential intervention opportunities by adherence to healthy lifestyles. Aging-related DNAm and altered gene expression might contribute to this biological association, which yielded insights into the etiology and potential therapeutic targets of CRC.

**Funding:**

The National Nature Science Foundation of China, Zhejiang Provincial Clinical Research Center for CANCER, and the National Institutes of Health.

**Research in context**

**Evidence before this study**

We searched PubMed to find studies examining the relationship of biological aging with colorectal cancer (CRC) by using terms “(colorectal cancer) AND ((biological aging) OR (leukocyte telomere length) OR (frailty) OR (PhenoAge) OR (homeostatic dysregulation) OR (Klemera-Doubal method biological age) OR (epigenetic clock))”. Emerging evidence has shown that biological aging is linked to CRC risk. However, the mechanisms underlying the association between biological aging and CRC and potential intervention measures are not fully understood.

**Added value of this study**

Using a comprehensive analysis strategy, we provided multidimensional evidence for the positive association of biological aging with the risk of CRC and its subgroups, especially extending the association evidence for early-onset CRC. This study uncovered that accelerated biological aging mediated the adverse effect of unhealthy lifestyle and its components (smoking, alcohol drinking, unhealthy diet, inadequate physical activity) on CRC. Furthermore, our findings highlighted that modified DNA methylation at aging-related CpGs and altered gene (i.e., *TNF*, *BICC1*, *DIP2B*, *NCF2*) expression patterns contributed to this biological association.

**Implications of all the available evidence**

Adherence to a healthy lifestyle could be a promising interventional measure to delay biological aging and subsequently mitigate the risk of CRC. The adverse association between biological aging and CRC might be underpinned by modified DNA methylation at aging-related CpGs and altered gene expression patterns, which provided insights into the etiological pathways of biological aging in the development of CRC and potential targets for CRC treatment.

## Introduction

Colorectal cancer (CRC) was the third most common malignancy and the second leading cause of cancer mortality worldwide in 2022, with more than 1.9 million incidence cases and 0.9 million deaths.^1^ The risk of developing CRC increases dramatically with age, and aging is thought to be one of the primary risk factors for CRC.^2^ Yet, CRC risk varies substantially between individuals, even within the same chronological age group, and the incidence of early-onset CRC (EOCRC) (diagnosed before 50 years of age) has gradually increased in recent years,^3^ which may reflect heterogeneity in underlying chronological aging.

Recently, biological aging, defined as an increased state of cellular vulnerability and measured from biological and physiological factors, has been proposed and used as a better tool to understand and predict disease than chronological aging.^4^^,^^5^ Several common measurements of biological aging include individual biomarkers, such as telomere length,^6^ algorithms that integrate information across epigenetic, other omics, or clinical biomarkers, such as PhenoAge algorithms, Klemera-Doubal method biological age (KDM-BA), and homeostatic dysregulation (HD) score,^7^, ^8^, ^9^, ^10^ as well as clinical assessments, such as frailty.^11^ Emerging evidence has shown that biological aging is associated with increased CRC risk.^12^, ^13^, ^14^, ^15^, ^16^ However, the underlying mechanisms of biological aging on CRC development are not fully understood.

Epigenetic modifications such as DNA methylation (DNAm) are thought to be the most accurate molecular readouts of biological aging,^17^^,^^18^ and previous research has revealed alterations in DNAm to be closely related to the development of CRC.^19^^,^^20^ A deep investigation of the relationship between biological aging, its fingerprints in blood DNAm and CRC may increase our understanding of the potential molecular pathways that underpin these associations. Additionally, biological aging and the development of CRC have been found to be strongly influenced by modifiable risk factors, such as diet, physical activity, alcohol drinking, and smoking.^17^^,^^18^^,^^21^ Elucidating how biological aging contributes to the association between these modifiable risk factors and CRC risk may represent intervention opportunities, especially since current knowledge regarding the relationship between modifiable risk factors, biological aging and CRC is still limited.

Here, we first utilized a longitudinal study design to evaluate the association between biological aging, modifiable risk factors (i.e., diet, physical activity, smoking, and alcohol drinking, as well as a lifestyle index of these factors combined), and the incidence of CRC and its diagnosis age-based or anatomical subgroups (i.e., EOCRC, late-onset CRC [LOCRC, diagnosed ≥50 years of age], colon cancer [proximal, distal], rectum cancer). We then assessed how biological aging contributes to the association between modifiable risk factors and CRC. In addition, we evaluated the relationship of biological aging with CRC risk from an epigenetic perspective (i.e., epigenetic aging clocks) by employing Mendelian randomization (MR) analysis. Finally, a three-step MR analysis and colocalization analysis were performed between aging-related DNAm (i.e., CpG sites), gene expression, and CRC to identify the key CpGs sites, genes and possible molecular pathways underlying aging and CRC, which may represent potential therapeutic targets.

## Methods

### Ethics

In this study, the individual-level data were from the UK Biobank (UKBB), which has received approval from the North West Multicenter Research Ethical Committee (11/NW/0382). Written informed consent was obtained from all participants.

### Statistics

#### Population-based longitudinal cohort study

##### Study population

**Fig. 1:**
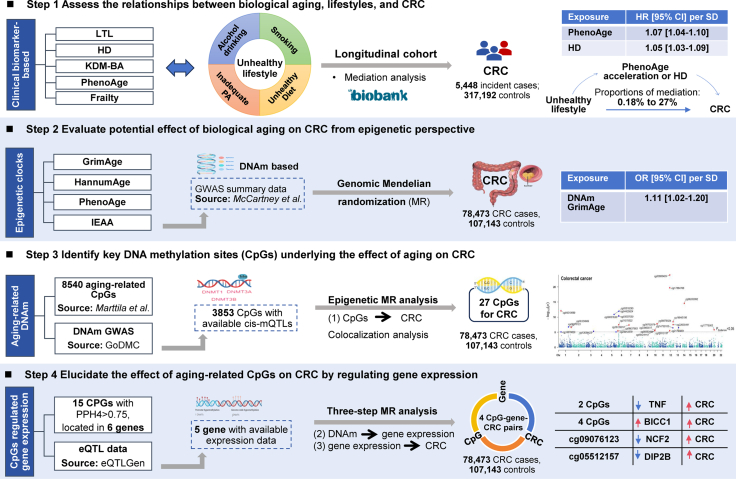
Flowchart of the study design. CRC, colorectal cancer; LTL, leukocyte telomere length; HD, homeostatic dysregulation; KDM-BA, Klemera-Doubal method biological age; PA, physical activity; DNAm, DNA methylation; IEAA, Intrinsic epigenetic age acceleration; GWAS, genome-wide association study; PPH4, posteriori probability for H4; mQTL, methylation quantitative trait locus; eQTL, expression quantitative trait locus; HR, hazard ratio; OR, odds ratio; CI, confidence interval.

The UKBB is a prospective research cohort that recruited more than 500,000 participants from 22 centers in the United Kingdom from 2006 to 2010. Detailed information on the UKBB study has been described elsewhere.^22^ After excluding participants with incomplete information for evaluating biological aging, non-white participants, and with cancer at recruitment, a total of 322,640 individuals (5448 incident CRC cases and 317,192 controls) aged 38–73 years remained, including 78 EOCRC, 5370 LOCRC, 3678 colon cancer (2158 proximal colon cancer, 1843 distal colon cancer), and 1770 rectal cancer incident cases, respectively (Supplementary Figure 1). An overview of the study design is shown in Fig. 1.

##### Assessment of biological aging based on clinical indicators or biomarkers

To quantify biological aging, we employed a number of multidimensional indicators including: individual biomarker-based leukocyte telomere length (LTL),^23^ clinical indicators and biomarkers-based PhenoAge acceleration,^9^ KDM-BA acceleration^8^ and HD score,^10^ and clinical assessment-based frailty phenotype (FP).^11^ We calculated these biological aging indicators using well-established methods and formulas based on individual-level data of clinical assessments and biomarkers measured by UKBB.

Specifically, in UKBB, LTL was assessed by quantitative polymerase chain reaction technique and was quantified as a ratio of telomere repeats copy number to single gene copy number, and it was then loge-transformed and z-standardized. The specific details have been reported elsewhere.^23^ We calculated PhenoAge using chronological age and 9 blood chemistries, including alkaline phosphatase, albumin, C-reactive protein, creatinine, glucose, mean cell volume, red cell distribution width, white blood cell count, and lymphocyte proportion, with aging-related mortality as the output variable in training.^9^ KDM-BA was calculated from systolic blood pressure, forced expiratory volume in 1 s, and 7 blood chemistry parameters (i.e., alkaline phosphatase, albumin, blood urea nitrogen, C-reactive protein, creatinine, glycated hemoglobin, and total cholesterol), using chronological age as the output variable in training.^8^ HD score utilizes the Mahalanobis distance^10^ to assess how unusual an overall profile of biomarkers (i.e., albumin, alkaline phosphatase, C-reactive protein, total cholesterol, creatinine, glycated hemoglobin, systolic blood pressure, blood urea nitrogen, uric acid, lymphocyte percent, mean cell volume, white blood cell count) (Supplementary Methods) is for a given participant compared to a reference population, using the NHANES III nonpregnant participants aged 20–30 years as reference samples. It reflects the deviation of the person's physiology from the relatively young and healthy reference sample, implying the degree of multi-system physiological dysregulation that is a key biological mechanism of aging.^7^ The natural logarithm transformation was utilized to approximate normality. To quantify the deviation between biological age and chronological age, PhenoAge acceleration and KDM-BA acceleration were defined as the residual values calculated by regressing PhenoAge and KDM-BA values on chronological ages at the time of biomarker measurement, respectively.^24^ Residuals were not estimated for HD score because it was not an age measure.^15^ The R package ‘BioAge’^25^ was employed to calculate these indices of biological aging. Details of the measurements for biological aging are shown in Supplementary Methods and Supplementary Table 1. Frailty was measured using FP,^11^ which was evaluated using 5 criteria (i.e., grip strength, exhaustion, weight loss, physical activity, walking speed) (Supplementary Table 2).^26^ The FP score ranged from 0 to 5, with a higher score indicating more severe frailty. Participants were categorized into three groups (non-frail: FP score = 0, pre-frail: FP score≥1 to ≤2, and frail: FP score≥3), based on previously conducted studies.^11^^,^^26^

##### Assessment of modifiable risk factors

Diet (healthy; unhealthy), smoking status (current smoker; non-current smoker: never/former smoker), alcohol consumption status (heavy drinker: ≥14 g/d for female and ≥28 g/d for male; non-drinker/moderate drinker: <14 g/d for female and <28 g/d for male) based on the American Cancer Society guidelines for Cancer Prevention,^27^ and physical activity (adequate physical activity; inadequate physical activity) were collected in the UKBB. These factors were determined by participants answering a questionnaire at baseline. We further constructed a behavior-based lifestyle index by summing the number of healthy lifestyle factors (i.e., non-current smoker, non-drinker/moderate drinker, healthy diet, and adequate physical activity) to capture overall lifestyle habits/behaviors.^28^ The lifestyle index ranged from 0 to 4, with higher values indicating more adherence to healthy lifestyle practices. We subsequently categorized the lifestyle index into current healthy lifestyle (3–4 scores) and unhealthy lifestyle (0–2 scores). A detailed description of the construction criteria of these factors is shown in Supplementary Table 3.

##### Definition of outcome

The primary outcome was CRC diagnosis, which was defined as malignant neoplasms of the colon and rectum and coded using the International Classification of Diseases (ICD, ICD-9: 154.0, 154.1, 153; or ICD-10: C18–C20) in the UKBB by linkage to hospital data and cancer registry. We further defined individuals who were diagnosed with CRC before 50 years of age as EOCRC, and individuals who were diagnosed with CRC at 50 years old and more as late-onset CRC (LOCRC). The date of enrolment in the UKBB was considered as the start of the follow-up period, and follow-up was censored at the date of incident CRC, death, or date of final registry update (October 31, 2022), whichever came first.

##### Evaluation of the association between biological aging and onset of CRC

The baseline characteristics of participants were described as median (Q1-Q3) for continuous variables and number (percentage) for categorical variables, and the Wilcoxon rank sum test or Pearson's Chi-squared test was used to compare the difference between incident CRC cases and individuals without CRC.

Correlation between biological aging and chronological age was evaluated by Pearson correlation analysis. Spearman correlation analysis was also performed to check the robustness of the results. In the longitudinal analyses of the association between biological aging and CRC risk, Cox proportional hazards (CPH) models were used to estimate hazard ratios (HRs) and 95% confidence intervals (95% CIs), with follow-up time as the time scale. Sample size for CPH model was calculated by using ‘powerSurvEpi’ package.^29^ Refer to the previous publications (e.g., HR of PhenoAge on CRC = 1.09),^15^ the minimum sample size required was 75,325 (1272 CRC cases) to obtain a statistical power of 0.8 (alpha = 0.05, two-sided test). PH assumption was assessed by examining Schoenfeld residuals. The model was adjusted for age at recruitment, sex, education, smoking status, alcohol consumption, diet, physical activity, Townsend deprivation index (TDI), household income, body mass index (BMI), diabetes, hypertension and coronary artery disease (CVD). The definitions of these covariates are presented in Supplementary Table 4. For missing covariates, categorical variables were replaced with missing indicators, while continuous variables were replaced via multiple imputation with fully conditional specification methods.^30^ The associations of biological aging with EOCRC, LOCRC, colon cancer, proximal colon cancer, distal colon cancer, and rectal cancer were also evaluated. Stratified analysis by sex was then performed. Heterogeneity across diagnosis age-based CRC subgroups (EOCRC and LOCRC) or sex was tested to evaluate whether the biological aging-CRC association differed between EOCRC and LOCRC or between males and females. To assess the robustness of results, we further performed sensitivity analyses: 1) using CPH models with age as the time scale to better control for the effect of chronological age; 2) using a CPH competing risk model to control competing risks (non-CRC-related deaths) by employing the R package ‘tidycmprsk’^31^; 3) re-evaluating the association between biological aging and CRC after imputation of categorical missing covariates via multiple imputation with fully conditional specification methods^30^; 4) For associations of biological aging with CRC that failed in the PH assumption (Schoenfeld residuals test *P* < 0.05), we performed sensitivity CPH analysis across increasing yearly case follow-up times, in which cases diagnosed after the case follow-up interval were assigned as controls.^32^

##### Estimation of mediation effect of biological aging on lifestyle factors and CRC

To assess how biological aging contributes to the associations between modifiable risk factors and CRC, we calculated the mediation proportion of biological aging for the association of unhealthy lifestyle factors and lifestyle index with CRC. First, CRC incidence was regressed by unhealthy lifestyle factors, biological aging indicators, and confounders by fitting a parametric (Weibull) survival regression model. Then, biological aging indicators (the mediator) were regressed by unhealthy lifestyle factors respectively in multivariable linear models adjusting for confounders. Subsequently, the models were combined to measure the natural direct and indirect effects. The mediation proportion was estimated by the R package ‘mediation’.^33^ Given that measurement error in any of the exposure, mediator, or outcome variables could bias estimates of mediation effects, we further performed sensitivity analyses by implementing inverse probability weighting (IPW) in mediation models to assess robustness.^34^ In addition, an interaction analysis between unhealthy lifestyles and biological aging was performed to assess their combined effect on CRC risk. R version 4.2.2 was used to conduct data cleaning and statistical analyses. For correction of multiple testing, the statistical significance was defined as two-sided *P*-values <0.05/number of factors.

#### Summary data-based Mendelian randomization study

##### Data sources

We further employed DNAm-based biological aging phenotypes. Genetic instruments for four epigenetic clocks were obtained from a genome-wide association study (GWAS) conducted among participants of European ancestries, including DNAm PhenoAge acceleration (DNAm PhenoAge, n = 34,463), DNAm GrimAge acceleration (DNAm GrimAge, n = 34,467), DNAm Hannum age acceleration (DNAm Hannum, n = 34,449), and intrinsic epigenetic age acceleration (DNAm IEAA, n = 34,461)^35^ (Supplementary Table 5). Aging-related CpG sites were derived from the aging epigenome-wide association studies (EWAS).^36^ Methylation quantitative trait loci (mQTLs) associated with aging-related CpGs were extracted from the Genetics of DNA Methylation Consortium (GoDMC) (n = 32,851).^37^ Expression quantitative trait loci (eQTLs) data for CpGs located genes were obtained from Expression Quantitative Trait Loci Genetics Portal (eQTLGen) (n = 31,684).^38^

Summary statistics of genetic associations for CRC, colon cancer, and rectal cancer were extracted from large-scale GWAS studies of CRC (78,473 cases, 107,143 controls),^39^ colon cancer (3793 cases, 410,350 controls),^40^ and rectal cancer (2091 cases, 410,350 controls),^40^ respectively. Supplementary Table 6 describes the specific sources of CRC data.

##### Evaluation of the association between epigenetic clocks and CRC

MR analyses were performed to evaluate the potential causal relationship of four epigenetic clocks (i.e., DNAm GrimAge, DNAm Hannum, DNAm PhenoAge, DNAm IEAA) with the risk of CRC and its anatomical subgroups (i.e., colon cancer, rectal cancer), using the "TwoSampleMR" package.^41^ MR requires three assumptions: instrumental variables (IVs) 1) are associated with the exposure, 2) share no unmeasured cause with the outcome, and 3) are associated with the outcome only through exposure.^42^ Single nucleotide polymorphisms (SNPs) meeting the following criteria were used as IVs for the epigenetic aging clocks: 1) SNPs associated with an epigenetic aging clock at the genome-wide significant threshold (*P* < 5 × 10^−8^); 2) linkage disequilibrium (LD) clumping (r^2^ < 0.001 for SNPs within 10000 kb) was then performed to select independent IVs. The R^2^ and F-statistics were calculated to evaluate the strength of genetic instruments. Details of all IVs after matching and harmonization with CRC outcome data are presented in Supplementary Table 7. The proportion of the variability of the DNAm GrimAge explained by all independent genetic instruments (4 IVs) was 6%, 20% for DNAm Hannum (8 IVs), 41% for DNAm PhenoAge (11 IVs), and 61% for DNAm IEAA (22 IVs).

The inverse variance weighted (IVW) MR method with random-effects was adopted as the main analysis. Four additional methods (i.e., MR-Egger, simple mode, weighted mode, and weighted median) were performed as sensitivity analyses. For correction of multiple testing, the statistical significance was defined as the two-sided P-values <0.0125 (0.05/number of epigenetic clocks).

##### Estimation of the association between aging-related DNAm and CRC

A total of 8540 aging-related CpG sites were reported by a previous aging EWAS.^36^ Of them, 3853 aging-related CpG sites had available cis-methylation quantitative trait loci (mQTLs) and these cis-mQTLs were extracted from the GoDMC^37^ (Supplementary Table 8). The selection criteria of IVs for CpGs are the same as described above. After matching and harmonization with the outcome data, 3453 CpGs for CRC, 3483 CpGs for colon cancer, and 3488 CpGs for rectal cancer were included in the epigenetic MR analysis, respectively.

When CpGs had only one instrument, the Wald ratio method was employed to evaluate the log odds change in CRC risk for per standard deviation (SD) increment of DNA methylation level as proxied by the IV. When CpGs had two or more genetic instruments, the IVW method was used. Additional models, including MR-Egger, simple mode, weighted median, and weighted mode, were used to account for potential horizontal pleiotropy. The Bonferroni correction method was utilized for correction of multiple testing, with a *P*_Bonferroni_ (the original p-value multiplied by the number of comparisons) < 0.05 (equivalent to the original p-value <1.3 × 10^−5^) as the statistically significant level.

##### Prioritization of differentially methylated CpGs underlying the effect of biological aging on CRC

For statistically significant CpGs in the epigenetic MR, we further performed colocalization analyses using the "coloc" package^43^ to assess whether the two associated signals (CpGs and CRC risk) were driven by common causal genetic variables to distinguish the confounding of LD.^44^^,^^45^ The colocalization analysis was conducted under five hypotheses: 1) H0, there was no causal genetic variant for both phenotypes (CpG and CRC) in the genomic locus; 2) and 3) there was one causal variant for CpG only (H1) or CRC only (H2), respectively; 4) H3, there were two distinct causal variants for CpG and CRC; 5) H4, there was a shared causal genetic variant for CpG and CRC. Each hypothesis was quantified by the posterior probability, and a posteriori probability (PPH4) ≥0.8 was considered high evidence of colocalization. We used default parameters and prior definitions. For each CpG, SNPs within ±500 kb of the mQTL were included. For CpGs with more than one mQTL, colocalization analysis was performed based on each mQTL, respectively, and the mQTL with the largest PPH4 was reported.

##### Estimating the association between aging-related CpGs on CRC by regulating gene expression

DNA methylation is thought to regulate gene expression. To explore how aging-related CpGs affected the CRC risk, we further performed a three-step MR analysis to assess the comprehensive relationships between identified aging-related CpGs, gene expression, and CRC risk. First, we evaluated the association between aging-related CpGs and CRC risk, which had been described above (step 1). For identified statistically significant CpGs, we further assessed the association between these CpGs and the expression of their mapped genes in the blood (step 2). Last, we estimated the association between gene expression and CRC risk (step 3). In step 2, the mQTLs of identified aging-related CpGs were used as exposure data, and the GWAS summary data of gene expression for CpG-mapped genes from the eQTLGen^38^ were used as outcome data. In step 3, eQTLs data for CpG mapped gene were used as exposure data and CRC GWAS summary data as outcome data based on summary-data-based MR.^46^

### Role of funders

The funders had no role in study design, data collection and analysis, decision to publish, or preparation of the manuscript.

## Results

### Baseline characteristics

In this longitudinal cohort of 322,640 participants, during a median follow-up time of 13.80 (Q1-Q3, 13.10–14.46) years, 5448 participants were diagnosed with incident CRC (78 EOCRC, 5370 LOCRC) (Supplementary Table 9). The median age at baseline of all participants included was 58 (Q1-Q3, 50–63) years (43 [Q1-Q3, 42–45] years for EOCRC, 62 [Q1-Q3, 57–66] years for LOCRC), and 151,277 (47%) were female. In all participants, 10% were current smokers, 29% were heavy drinkers, 34% consumed an unhealthy diet, and 26% had inadequate physical activity at baseline, and 4.5% had diabetes, 27% hypertension, or 9.5% CAD at baseline. Compared to individuals without CRC, incident CRC cases were more biologically older at baseline.

### Longitudinal association between biological aging and CRC incidence

Statistically significant correlation between biological aging measures and chronological age was observed (*P* < 0.001), except for PhenoAge acceleration, which was defined as the residual value calculated by regressing PhenoAge on chronological age (Supplementary Figure 2). In the multivariate adjustment model (Table 1), PhenoAge acceleration was associated with a higher risk of CRC (HR [95% CI]: 1.07 [1.04–1.10] per SD increment, *P* = 5.00E–06) and its anatomical subgroups (colon cancer, proximal colon cancer, and distal colon cancer). HD score was observed to be associated with increased risk of CRC (HR [95% CI]: 1.05 [1.03–1.09] per SD increment, *P* = 1.89E–04) and its anatomical subgroups (colon cancer and proximal colon cancer). None of these scores was associated with rectal cancer. In stratified analysis by age at diagnosis, PhenoAge acceleration and HD score were associated with an increased risk of both EOCRC (HR [95% CI] per SD increment: 1.29 [1.07–1.55], *P* = 8.35E–03 for PhenoAge acceleration; 1.35 [1.08–1.69], *P* = 8.77E–03 for HD score) and LOCRC (HR [95% CI] per SD increment: 1.06 [1.03–1.09], *P* = 2.78E–05 for PhenoAge acceleration; 1.05 [1.02–1.08], *P* = 5.70E–04 for HD score), and showed a stronger association effect on EOCRC than LOCRC (*P*_heterogeneity_<0.05) (Supplementary Table 10). In stratified analysis by sex, the association between PhenoAge acceleration and CRC was stronger in males than in females (Supplementary Table 11). Similar results were observed in CPH model with age as the time scale (Supplementary Table 12) and competing risk model (Supplementary Table 13) and after imputation of categorical missing covariates (Supplementary Table 14). For the associations (i.e., HD score or PhenoAge acceleration and CRC) failed the Cox PH assumption (Schoenfeld residual test *P* < 0.05) (Supplementary Table 15), we further performed sensitivity analysis and found a statistically significant but diminishing association across increasing yearly case follow-up times (Supplementary Table 16). The strongest association was observed in the first year of case follow-up (HR [95% CI] per SD increment: 1.47 [1.31–1.64] for HD score and 1.53 [1.40–1.67] for PhenoAge acceleration).

**Table 1:** Associations of biological aging with incident colorectal cancer and diagnosis age-based or anatomical subgroups.

Biological aging	CRC			EOCRC			LOCRC			Colon			Proximal			Distal			Rectum		
	case	HR (95% CI)	*P*	case	HR (95% CI)	*P*	case	HR (95% CI)	*P*	case	HR (95% CI)	*P*	case	HR (95% CI)	*P*	case	HR (95% CI)	*P*	case	HR (95% CI)	*P*
**LTL**																					
Continuous, per SD increment	5448	1.02 (0.99–1.05)	0.15	78	1.11 (0.89–1.40)	0.35	5370	1.02 (0.99–1.05)	0.17	3678	1.02 (0.99–1.05)	0.25	2158	1.01 (0.96–1.05)	0.79	1843	1.04 (1.00–1.09)	0.07	1770	1.02 (0.97–1.07)	0.39
**HD score**																					
Continuous, per SD increment	5448	1.05 (1.03–1.09)	1.89E–04	78	1.35 (1.08–1.69)	8.77E–03	5370	1.05 (1.02–1.08)	5.70E–04	3678	1.06 (1.02–1.09)	1.25E–03	2158	1.08 (1.03–1.13)	5.18E–04	1843	1.05 (1.00–1.10)	0.05	1770	1.05 (1.00–1.10)	0.06
**KDM-BA acceleration**																					
Continuous, per SD increment	5448	0.98 (0.96–1.01)	0.16	78	1.22 (0.94–1.58)	0.14	5370	0.98 (0.95–1.01)	0.12	3678	0.97 (0.94–1.01)	0.12	2158	0.97 (0.93–1.01)	0.20	1843	0.97 (0.93–1.02)	0.24	1770	0.99 (0.95–1.04)	0.83
**PhenoAge acceleration**																					
Continuous, per SD increment	5448	1.07 (1.04–1.10)	5.00E–06	78	1.29 (1.07–1.55)	8.35E–03	5370	1.06 (1.03–1.09)	2.78E–05	3678	1.08 (1.04–1.11)	1.60E–05	2158	1.09 (1.05–1.14)	4.89E–05	1843	1.07 (1.02–1.13)	3.62E–03	1770	1.05 (1.00–1.10)	0.07
**Frailty phenotype**																					
Continuous, per score increment	5448	0.99 (0.95–1.02)	0.50	78	1.07 (0.78–1.48)	0.67	5370	0.99 (0.95–1.02)	0.45	3678	0.98 (0.94–1.03)	0.49	2158	1.02 (0.96–1.07)	0.58	1843	0.96 (0.90–1.02)	0.16	1770	0.99 (0.93–1.06)	0.79
Non-frail [0]	2902	Ref		45	Ref		2857	Ref		1926	Ref		1103	Ref		994	Ref		976	Ref	
Pre-frail [1–2]	2389	1.00 (0.94–1.06)	0.96	31	1.20 (0.75–1.93)	0.44	2358	0.99 (0.94–1.05)	0.85	1641	1.01 (0.94–1.08)	0.88	980	1.03 (0.94–1.12)	0.52	805	0.99 (0.90–1.09)	0.90	748	0.98 (0.89–1.09)	0.74
Frail [3–5]	157	0.95 (0.81–1.12)	0.56	2	1.78 (0.40–7.83)	0.45	155	0.95 (0.80–1.12)	0.51	111	0.94 (0.77–1.14)	0.53	75	1.08 (0.85–1.38)	0.53	44	0.79 (0.58–1.08)	0.13	46	0.98 (0.72–1.33)	0.87

Results were derived from Cox proportional hazards regression analysis, with follow-up time as the time scale. Model was adjusted for age at recruitment, sex, education, smoking status, alcohol consumption, diet, physical activity, Townsend deprivation index, household income, body mass index, history of diabetes, history of hypertension, and history of coronary artery disease.

LTL, leukocyte telomere length; HD, homeostatic dysregulation; KDM-BA, Klemera-Doubal method biological age; CRC, colorectal cancer; EOCRC, early-onset CRC; LOCRC, late-onset CRC; SD, Standard deviation; Ref, reference; HR, hazard ratio; CI, confidence interval.

### The role of biological aging on the association of lifestyle factors with CRC

After multivariate adjustment (Supplementary Table 17), current smoking (HR [95% CI]: 1.13 [1.04–1.24], comparing with non-current smoker), alcohol drinking (HR [95% CI]: 1.24 [1.17–1.32] for heavy drinker vs. non-drinker/moderate drinker; 1.11 [1.09–1.14] for per SD increment), inadequate physical activity (HR [95% CI]: 1.10 [1.03–1.16], comparing with adequate physical activity), unhealthy diet (HR [95% CI]: 1.12 [1.06–1.19], comparing with healthy diet), and unhealthy lifestyle (HR [95% CI]: 1.27 [1.19–1.35], comparing with healthy lifestyle) were associated with increased risk of CRC. Similar results were observed for LOCRC, colon cancer, and proximal colon cancer, but not for EOCRC, and only alcohol drinking, unhealthy diet, and unhealthy lifestyle were associated with increased risk of distal colon cancer and rectal cancer. Furthermore, we found that these unhealthy lifestyle risk factors for CRC were also associated with higher PhenoAge acceleration and HD score (beta>0, *P* < 0.01), except for alcohol consumption and HD score (Table 2, Supplementary Table 18). The associations between unhealthy lifestyle factors (smoking, alcohol drinking, unhealthy diet, inadequate physical activity, unhealthy lifestyle) and CRC were partly mediated by increased PhenoAge acceleration or HD score, with proportions of mediation ranging from 0.18% to 27.00% (*P* < 2.00E-16). For instance, the increased PhenoAge acceleration mediated 27.00% of the adverse effect of smoking on CRC. Similar mediation results were observed in sensitivity analyses (Supplementary Table 19). No statistically significant interaction between unhealthy lifestyles and biological aging on CRC risk was found (Supplementary Table 20).

**Table 2:** Mediation analysis of the association between lifestyle factors (exposure) and colorectal cancer (outcome), with biological aging as the mediator.

Lifestyle factors (exposure)	Mediator	Lifestyle-CRC		Lifestyle-Biological aging		Biological aging-CRC		Mediation effect	
		HR (95% CI)^a^	*P*^a^	Beta (95% CI)^b^	*P*^b^	HR (95% CI)^c^	*P*^c^	Mediated proportion^d^	*P*^d^
**Smoking status**	PhenoAge acceleration					1.07 (1.04–1.10)	5.00E-06		
Current smoker vs. Non-current smoker (never/former)		1.13 (1.04–1.24)	6.42E–03	0.48 (0.47, 0.49)	<1.00E–300			27.00%	<2.00E–16
**Alcohol drinking**									
Heavy drinker vs. Non-drinker/moderate drinker		1.28 (1.20–1.37)	7.84E–14						
Per SD increment		1.11 (1.09–1.14)	3.36E–26	0.01 (0.00, 0.01)	3.50E–04			0.41%	<2.00E–16
**Physical activity**									
Inadequate physical activity vs. Adequate physical activity		1.10 (1.03–1.16)	2.32E–03	0.08 (0.08, 0.09)	2.71E–117			6.68%	<2.00E–16
**Diet**									
Unhealthy vs. Healthy		1.12 (1.06–1.19)	5.30E–05	0.17 (0.17, 0.18)	<1.00E–300			10.30%	<2.00E–16
**Lifestyle**									
Unhealthy vs. Healthy		1.27 (1.19–1.35)	5.28E–15	0.22 (0.21, 0.23)	<1.00E–300			6.03%	<2.00E–16
**Smoking status**	HD score					1.05 (1.03–1.09)	1.89E–04		
Current smoker vs. Non-current smoker (never/former)		1.13 (1.04–1.24)	6.42E–03	0.22 (0.21, 0.22)	<1.00E–300			1.63%	<2.00E–16
**Physical activity**									
Inadequate physical activity vs. Adequate physical activity		1.10 (1.03–1.16)	2.32E–03	0.08 (0.07, 0.08)	1.95E–100			4.37%	<2.00E–16
**Diet**									
Unhealthy vs. Healthy		1.12 (1.06–1.19)	5.30E–05	0.05 (0.04, 0.06)	5.50E–52			2.26%	<2.00E–16
**Lifestyle**									
Unhealthy vs. Healthy		1.27 (1.19–1.35)	5.28E–15	0.01 (0.00, 0.02)	9.27E–03			0.18%	<2.00E–16

CRC, colorectal cancer; HR, hazard ratio; CI, confidence interval; HD, homeostatic dysregulation.

a Results were derived from Cox proportional hazards regression analysis, with follow-up time as the time scale. Model was adjusted for age at recruitment, sex, education, Townsend deprivation index, household income, body mass index, history of diabetes, history of hypertension, and history of coronary artery disease.

b Results were derived from linear regression analysis. Model was adjusted for age at recruitment, sex, education, Townsend deprivation index, household income, body mass index, history of diabetes, history of hypertension, and history of coronary artery disease.

c Results were derived from Cox proportional hazards regression analysis, with follow-up time as time scale. Model was adjusted for age at recruitment, sex, education, smoking status, alcohol consumption, diet, physical activity, Townsend deprivation index, household income, body mass index, history of diabetes, history of hypertension, and history of coronary artery disease.

d Results were derived from mediation analysis based on linear regression and parametric (Weibull) survival regression model. Model was adjusted the same list of covariates as detailed above.

### Genomic MR identified the association of epigenetic clocks with CRC risk

**Fig. 2:**
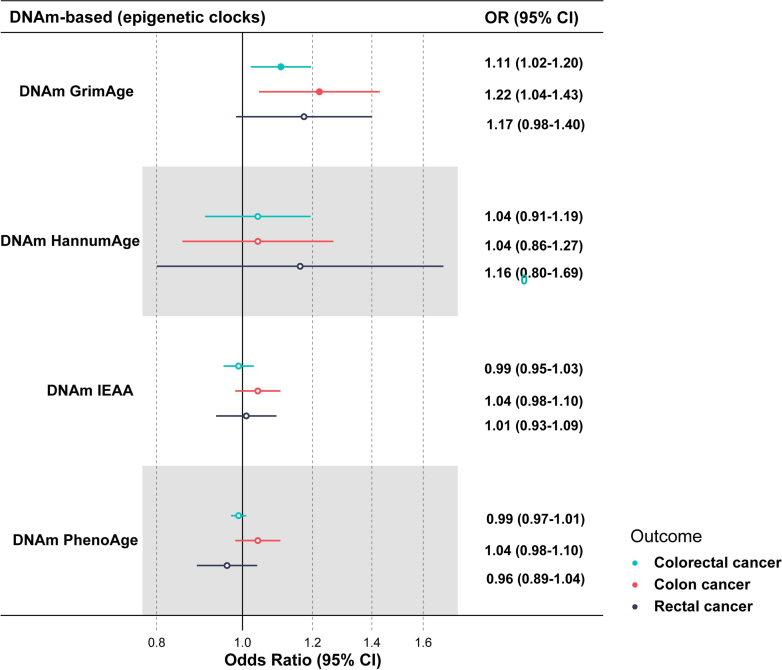
Mendelian randomization results of the association of epigenetic clocks with colorectal cancer, colon cancer, and rectal cancer. The center of the line represents the estimated OR, and the line represents the 95% CI. Solid circle indicates the association with *P* < 0.05. Here, OR means the odd ratio of outcome per SD increment in genetically predicted levels of the epigenetic clock. The sample sizes of exposure datasets were 34,467 for DNAm GrimAge acceleration, 34,449 for DNAm Hannum age acceleration (HannumAge), 34,461 for intrinsic epigenetic age acceleration (IEAA), and 34,463 for DNAm PhenoAge acceleration. The sample sizes of outcome datasets were 185,616 (78,473 cases, 107,143 controls) for colorectal cancer, 414,143 (3793 cases, 410,350 controls) for colon cancer, and 412,441 (2091 cases, 410,350 controls) for rectal cancer. DNAm, DNA methylation; OR, Odds ratio; CI, confidence interval.

To understand the relationship of biological aging with CRC from an epigenetic perspective, four epigenetic clocks were included in the genomic MR. The F-statistics for all IVs were larger than the conventional value of 10, suggesting that the IV were sufficiently strong (Supplementary Table 7). DNAm GrimAge was found to be positively associated with the risk of CRC (OR [95% CI] per SD increment: 1.11 [1.02–1.20], *P* = 0.018) with nominal significance and colon cancer (OR [95% CI] per SD increment: 1.22 [1.04–1.43], *P* = 0.007) passing multiple testing correction (Fig. 2, Supplementary Table 21). Whereas, no significant association between DNAm IEAA, DNAm PhenoAge, or DNAm Hannum and CRC was found. There was no detectable evidence of horizontal pleiotropy according to the MR Egger intercept test (*P*_pleiotropy_>0.05), and no evidence of heterogeneity was observed (*P*_heterogeneity_>0.05) (Supplementary Table 21).

### Epigenetic MR and colocalization revealed 15 aging-related DNAm sites for CRC

**Fig. 3:**
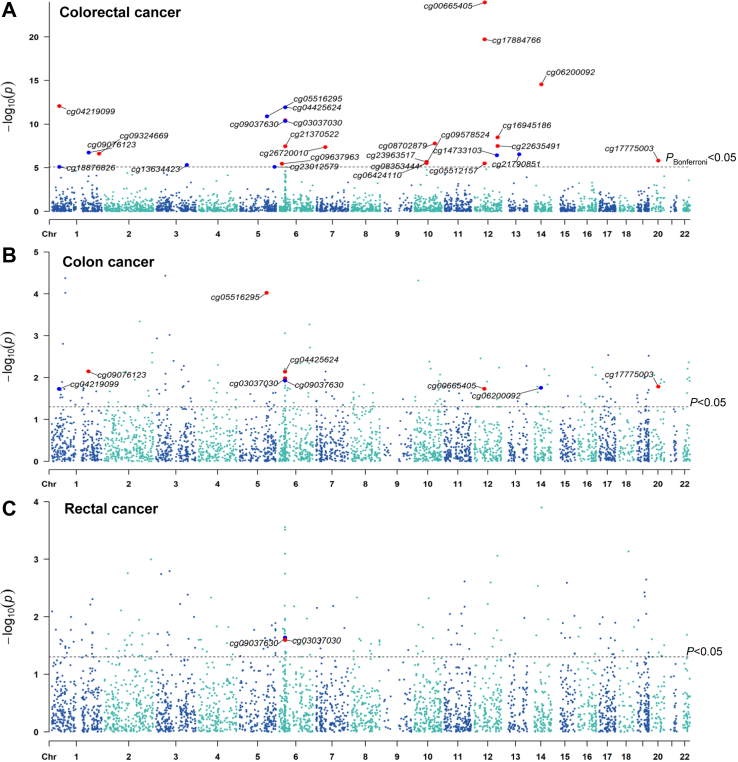
Manhattan plot showing results from epigenetic Mendelian randomization for colorectal cancer (A), colon cancer (B) and rectal cancer (C). The significant CpGs for CRC were labeled (*P*_Bonferroni_<0.05). The blue dots indicate inverse association, and red dots indicate positive association. The sample size of the exposure (methylation quantitative trait loci) dataset was 32,851. The sample sizes of outcome datasets were 185,616 (78,473 cases, 107,143 controls) for colorectal cancer, 414,143 (3793 cases, 410,350 controls) for colon cancer, and 412,441 (2091 cases, 410,350 controls) for rectal cancer.

In the epigenetic MR, altered methylation at 27 aging-related CpG sites was found to be associated with CRC risk (*P*_Bonferroni_<0.05), including 16 positive associations and 11 inverse associations (Fig. 3, Supplementary Table 22). 23 CpGs had overlapping SNPs in the same genomic coordinate, but none of these SNPs are mQTLs of these CpGs or have been reported to be associated with CRC risk (Supplementary Table 22). Among them, altered methylation at 9 aging-related CpGs (i.e., cg0065405, cg06200092, cg04219099, cg04425624, cg05516295, cg09037630, cg03037030, cg09076123, cg17775003) and 2 CpGs (i.e., cg09037630, cg03037030) were also associated with the risk of colon cancer and rectal cancer at the nominal significance level (*P* < 0.05), respectively, with consistent effect direction. No evidence of heterogeneity and pleiotropy was observed (*P*_heterogeneity_>0.05, *P*_pleiotropy_>0.05) (Supplementary Tables 23–25). 15 CpGs were further supported by high colocalization evidence (PPH4>0.8) (Supplementary Table 26), suggesting a high probability for a shared genetic variant for aging-related CpGs and CRC risk. Of them, 10 aging-related CpG sites were mapped to 6 genes (i.e., *TNF*, *BICC1*, *TBX3*, *SUCNR1*, *NCF2*, *DIP2B*), and the altered methylation of cg03037030 located in *TNF* was associated with the risk of CRC, colon cancer, and rectal cancer, with consistent effect direction (Fig. 3, Supplementary Table 22).

Three-step MR indicated the effect of aging-related CpGs on CRC by regulating gene expression.

To explore how aging-related CpGs affected the CRC risk, we further performed MR analysis to investigate the relationship between the altered methylation at the identified aging-related CpGs and the expression level of five of their mapped genes with available GWAS summary data of gene expression. We found that the altered methylation at cg04425624 and cg03037030 (*TNF*), cg13634423 (*SUCNR1*), cg08353444, cg23963517, cg06424110, and cg09578524 (*BICC1*), cg09076123 (*NCF2*), and cg05512157 (*DIP2B*) were associated with the expression of their mapped genes (*P* < 0.05) (Table 3). The genetically determined blood expression levels of 4 (*TNF, BICC1, NCF2,* and *DIP2B*) of 5 mapped genes were also significantly associated with CRC risk (*P* < 0.05). Specifically, the associations of the methylation at cg04425624 and cg03037030, cg09076123, and cg05512157 with CRC might be mediated by decreasing the expression level of their located gene *TNF*, *NCF2*, and *DIP2B*, respectively. The associations of the methylation at 4 CpGs (cg08353444, cg23963517, cg06424110, cg09578524) with CRC might be mediated by increasing the expression level of their located gene *BICC1*.

**Table 3:** The comprehensive relationships between aging-related DNA methylation, gene expression, and colorectal cancer risk.

Aging-related CpG sites	Mapped gene	CpG-CRC				CpG-Gene expression			Gene expression-CRC		
		beta	se	*P*	PPH4	beta	se	*P*	beta	se	*P*
cg04425624	TNF	0.22	0.03	1.23E–12	0.99	−1.21	0.35	5.98E–04	−0.17	0.03	1.87E–10
cg03037030	TNF	0.39	0.06	4.42E–11	0.98	−2.33	0.10	1.36E–131	−0.17	0.03	1.87E–10
cg08353444	BICC1	0.12	0.02	2.10E–06	0.90	0.62	0.03	1.79E–82	0.22	0.05	5.27E–06
cg23963517	BICC1	0.14	0.03	2.10E–06	0.90	0.64	0.03	2.35E–82	0.22	0.05	5.27E–06
cg09578524	BICC1	0.19	0.04	2.82E–06	0.89	0.51	0.03	2.35E–82	0.22	0.05	5.27E–06
cg06424110	BICC1	0.14	0.03	2.98E–06	0.89	0.84	0.04	6.26E–83	0.22	0.05	5.27E–06
cg05512157	DIP2B	0.81	0.17	3.16E–06	0.80	−0.84	0.09	1.11E–22	−0.27	0.06	2.03E–05
cg09076123	NCF2	0.31	0.06	1.82E–07	0.84	−0.95	0.07	2.33E–48	−0.27	0.05	4.09E–07
cg13634423	SUCNR1	0.24	0.05	4.87E–06	0.98	0.11	0.06	0.05	−0.06	0.10	0.60

CRC, colorectal cancer.

## Discussion

In this study, we integrated an observational with a MR analysis to provide a comprehensive investigation regarding the relationship between multidimensional biological aging, unhealthy lifestyles, aging-related DNAm, gene expression, and CRC risk. Our findings showed that biological aging (mainly PhenoAge acceleration and HD score) was associated with increased risk of CRC and its subgroups, especially with EOCRC. As has been observed previously,^2^ we also found that an unhealthy lifestyle (i.e., currently smoke, alcohol drinking, inadequate physically activity, and consume an unhealthy diet) overall, as well as for each lifestyle factor individually, were associated with a higher risk of CRC and these associations were partly mediated by biological aging acceleration. These results suggested that adherence to a healthy lifestyle might hinder biological aging acceleration and subsequently decrease the risk of CRC. From the epigenetic perspective, our findings highlighted that genetic susceptibility to DNAm GrimAge was associated with a higher risk of CRC, and modified DNA methylation at aging-related CpGs and altered gene (i.e., *TNF*, *BICC1*, *DIP2B, NCF2*) expression patterns contributed to this biological association.

Aging is thought to be closely linked to cancer development.^47^ We evaluated the relationship between biological aging and CRC risk from multiple dimensions and highlighted the positive association of HD score and PhenoAge acceleration with CRC risk. The HD score and PhenoAge acceleration seemed to show a stronger effect on EOCRC. Differences in biological aging, while likely the result of different genetics, lifestyles or other factors, may help to partly explain the phenomenon that some young adults are more likely to develop CRC than their peers of the same chronological age. It implies individuals with accelerated biological aging may be a higher-risk subpopulation of developing CRC, with the potential to benefit most from health intervention. Biological aging is thought to serve as a promising candidate biomarker for predicting CRC risk and improving risk stratification.^48^ A longitudinal cohort study also found that the clinical biomarker-based HD score and PhenoAge acceleration were significantly associated with a higher risk of CRC.^15^ HD score implies multi-system physiological dysregulation^10^^,^^25^ that involves increased vulnerability, chronic inflammation, and metabolic dysregulation,^49^ which may contribute to the development of CRC. PhenoAge, built using mortality (including deaths from malignant neoplasms) as output, can capture the risk of mortality, which may explain the detection ability of PhenoAge acceleration on the incidence of CRC with a relatively high mortality rate. Furthermore, another longitudinal study reported a positive association between DNAm-based PhenoAge and CRC.^16^ However, no evidence of DNAm PhenoAge on CRC risk was observed in the current MR analysis, but we observed a positive association of DNAm GrimAge with CRC risk, consistent with the findings of a previous MR study.^13^ DNAm GrimAge, derived from sex, chronological age, and DNAm-based proxies for smoking and several plasma protein levels, with all-cause mortality as output, has been reported to stand out from other epigenetic clocks in predicting mortality.^50^ It is understandable that the clock can detect the incidence of CRC with a relatively high mortality rate. Similar results have been observed in breast cancer. A longitudinal cohort study reported that clinical biomarker-based PhenoAge was positively associated with breast cancer risk,^51^ whereas a null association between DNAm PhenoAge and breast cancer was observed in a pooled analysis of longitudinal studies.^52^ The difference in the components between clinical biomarker-based PhenoAge which was constructed by blood chemistries and chronological age (training outcome: mortality) and DNAm PhenoAge which was constructed by DNAm data (training outcome: PhenoAge) may contribute to the inconsistent findings. Overall, different aging phenotypes were trained on different indexes, tissues, and populations, which may capture distinct aging processes, pathways, and mechanisms, leading to differential associations with cancer risks.^53^

Different from chronological age, biological age can be influenced by modifiable risk factors such as diet, physical activity, and other behaviors.^17^^,^^18^ We constructed an integrated lifestyle index for diet, physical activity, smoking status, and alcohol consumption status, and observed that an unhealthy lifestyle index and several of its components individually (smoking, inadequate physical activity, unhealthy diet) were positively associated with PhenoAge acceleration and HD score. Further, a higher PhenoAge acceleration or HD score partly mediated the adverse effect of unhealthy lifestyle and its components on CRC, especially for smoking. Similarly, Harris, K. et al. found that tobacco use and no exercise were associated with faster biological aging across several aging clocks in a cohort study,^54^ and another cohort study reported that moderate to vigorous physical activity was associated with lower DunedinPACE scores (a DNAm-based clock).^55^ Adhering to a healthy lifestyle was found to help promote healthy aging and longevity,^56^ which might be attributed in part to the regulation effect of healthy diet and lifestyle on gut microbiome.^57^ Our findings supported the positive association of unhealthy lifestyles with biological aging and expanded the evidence on the combination of unhealthy lifestyles, accelerated aging, and CRC risk, which suggested potential intervention opportunities by adhering to healthy lifestyles to delay biological aging and decrease the risk of CRC. However, only a part of the effect of unhealthy lifestyles on CRC risk is explained by biological aging, and many intrinsic and other unknown mechanisms deserve further investigation.

Although the intricate details governing aging and cancer have not been revealed, epigenetic alteration is one of the primary hallmarks of both aging and cancer.^5^ In this study, we identified 15 aging-related CpGs for CRC risk and prioritized four potential regulatory genes (*TNF, BICC1, NCF2,* and *DIP2B*) underlying the association. *TNF* (tumor necrosis factor) encodes a multifunctional inflammatory cytokine originally identified for its ability to promote hemorrhagic necrosis of transplanted solid tumors and for its cytotoxic activity against tumor cell lines.^58^ *TNF* polymorphisms are thought to be closely linked to human aging and longevity.^59^^,^^60^ Studies also have reported the significant association between *TNF* polymorphisms and CRC.^61^ Coupling TNF with alpha(V) integrin ligands showed improved antineoplastic activity in animal experiments.^58^ Our findings indicated that the *TNF* gene expression might mediate the association of methylation at cg04425624 and cg03037030 with CRC. *DIP2B*, encoding a protein with a DNA methyltransferase 1 associated protein 1 binding domain, is thought to play an important role in DNA methylation machinery.^62^ Previous meta-GWAS and eQTL analyses have reported *DIP2B* as a susceptibility gene for CRC.^63^^,^^64^ Our findings expanded this evidence and indicated that a higher methylation level at cg05512157 of *DIP2B* was associated with a lower expression of *DIP2B* and a higher risk of CRC. *BICC1* encodes an RNA-binding protein that plays an important role in cell polarity regulation and signaling.^65^^,^^66^ While no direct evidence regarding *BICC1* and CRC has been reported, *BICC1* has been found to drive pancreatic cancer progression via inducing VEGF-independent angiogenesis and might serve as a promising anti-angiogenic therapeutic target.^66^ In addition, *BICC1* was highly expressed in gastric cancer and significantly associated with tumor grade.^67^ Similarly, our results suggested the carcinogenic effect of *BICC1* on CRC and *BICC1* expression might mediate the adverse effect of higher methylation at 4 CpGs of *BICC1* gene on CRC. Our findings also implied that higher methylation at cg09076123 of *NCF2* might decrease its expression level and promote CRC development. The protein encoded by *NCF2* is a component of NADPH oxidase, which is involved in the regulation of cellular oxidative stress, immune responses, and inflammatory responses in the tumor microenvironment.^68^^,^^69^ *NCF2* was identified as a potential suppresser gene for non-small cell lung cancer and was associated with better survival.^70^ The exact mechanism regarding the gene and CRC still needs further study.

This study has several strengths. Firstly, we systematically evaluated the relationships of biological aging with the risk of CRC and its subgroups from multiple dimensions considering modifiable risk factors, which helps to comprehensively understand the relationship of biological aging with CRC and also implies intervention opportunities by adhering to healthy lifestyles. We further evaluated the association of aging-related DNAm, gene expression and CRC to discover the key CpGs (genes) and elucidate possible molecular processes underlying aging and CRC, which provided insights into the etiological pathways of biological aging in the development of CRC and potential targets for CRC treatment. In addition, this study used an MR design and colocalization analysis based on well-designed and larger-scale GWAS data, which enhances statistical power and reduces the risk of confounding bias and reverse causality. However, this study also has several limitations. Firstly, the current study population consisted of individuals of European origin due to the small sample size for non-whites in UKBB and current uncertainty on analysis in pooled ethnic samples for biological aging markers.^54^ Therefore, the generalizability of the current findings to other ancestries or potential ethnic differences requires further assessment. Secondly, the statistical power may be low in stratified analyses due to the small number of cases, especially for EOCRC. Thirdly, lifestyle information was collected using self-reported questionnaires, which might result in misclassification. Almost all lifestyle factors had only a single measure at baseline. We could not capture the long-term lifestyle changes at earlier life stages and during follow-up, except for smoking (never/former/current). Fourthly, methylation levels from the array data could be influenced by overlapping SNPs. Most CRC-related CpG sites have overlapping SNPs. Although none of these SNPs are mQTLs of these CpGs or have been reported to be associated with CRC risk, suggesting that the current results are unlikely to be influenced by these overlapping SNPs, the interpretation of these findings should still be cautious. Fifth, at least nine epigenetic clocks have been developed.^71^ However, we only tested the association between four epigenetic clocks and CRC due to the lack of GWAS summary data for other epigenetic clocks. Similarly, due to the limited data in UKBB, we couldn't assess frailty based on more comprehensive methods (e.g., Essential Frailty Toolset, EFT). Future studies focusing on other epigenetic clocks and using EFT to assess frailty are needed after data is available. Sixth, the main biological aging indices and summary level data of aging-related CpGs were derived from blood, and DNAm in colorectal tissue was not evaluated due to the limited data. While blood tissue is usually a proxy for internal organs directly involved in the etiology of diseases and blood-based biomarkers are valuable due to their accessibility and potential for non-invasive screening, they may not fully reflect epigenetic changes occurring in colorectal tissue. Although previous studies have shown that blood-derived biological aging indices or DNAm exhibit consistency or a degree of conservation across multiple tissue types, including colon tissue,^9^^,^^72^ caution is needed in interpreting the current results as direct indicators of colorectal tissue changes. Therefore, our findings should be viewed as suggestive of systemic processes potentially associated with CRC risk rather than colorectal tissue-specific mechanisms. Future studies using colorectal tissue methylation data may be valuable to validate the association between aging-related CpGs and CRC and further assess the potential effect on EOCRC.

In conclusion, our study provided multidimensional evidence for the positive association of biological aging with CRC risk and implied potential intervention opportunities by adherence to healthy lifestyles to delay biological aging and decrease CRC risk. In addition, our findings highlighted that the adverse effect of biological aging on CRC might be underpinned by modified DNAm at aging-related CpGs and altered gene expression patterns, which provided insights into the etiology and potential therapeutic targets of CRC. Further studies among non-European populations based on precise measurements of lifestyle and colorectal tissue-derived biological aging indices or DNAm are needed to validate the current findings.

## Contributors

X.L., J.S.: conceptualization. J.S., M.L.: formal analysis, software, visualization, and writing – original draft. J.S., X–X.L., J-Y.Y., J-H.Z.: data curation, methodology, and data interpretation. X-Q.Z., Z-S.Y., H–S.J., Y-Q.T., Z-L.B.: project administration and investigation. X.L., H.W., Q.C., E.T., S.S–W, M-Y.S., M.D., D-F.Z.: resources, supervision, and writing – review & editing. X.L., X–X.L., and J-Y.Y. have accessed and verified the data. X.L. was responsible for the decision to submit the manuscript. All authors critically reviewed the manuscript and contributed important intellectual content. All authors have read and approved the final manuscript as submitted.

## Data sharing Statement

The results of this study are included in this published article and its supplementary information files. The UK Biobank is an open access resource and bona fide researchers can apply to utilize the UK Biobank dataset by registering and applying at http://ukbiobank.ac.uk/register-apply/. This study used the UK Biobank data under application number 66354. The GWAS summary data for CRC, colon cancer, and rectal cancer are available through the GWAS catalog (accession no. GCST90255675, GCST90011811, GCST90011810). The GWAS summary data of epigenetic clocks are available through the GWAS catalog (accession no. GCST90014292, GCST90014288, GCST90014290, GCST90014289). The mQTLs data from GoDMC are available at http://mqtldb.godmc.org.uk/. The eQTLs data from eQTLGen are available at https://www.eqtlgen.org/.

## Declaration of interests

The authors declare no potential conflicts of interest.

## References

[1] Bray F., Laversanne M., Sung H. (2024). Global cancer statistics 2022: GLOBOCAN estimates of incidence and mortality worldwide for 36 cancers in 185 countries. CA Cancer J Clin.

[2] Keum N., Giovannucci E. (2019). Global burden of colorectal cancer: emerging trends, risk factors and prevention strategies. Nat Rev Gastroenterol Hepatol.

[3] Sinicrope F.A. (2022). Increasing incidence of early-onset colorectal cancer. N Engl J Med.

[4] Maugeri A., Barchitta M., Magnano San Lio R., Li Destri G., Agodi A., Basile G. (2020). Epigenetic aging and colorectal cancer: state of the art and perspectives for future research. Int J Mol Sci.

[5] López-Otín C., Blasco M.A., Partridge L., Serrano M., Kroemer G. (2023). Hallmarks of aging: an expanding universe. Cell.

[6] Blackburn E.H., Epel E.S., Lin J. (2015). Human telomere biology: a contributory and interactive factor in aging, disease risks, and protection. Science.

[7] Li Q., Wang S., Milot E. (2015). Homeostatic dysregulation proceeds in parallel in multiple physiological systems. Aging Cell.

[8] Klemera P., Doubal S. (2006). A new approach to the concept and computation of biological age. Mech Ageing Dev.

[9] Levine M.E., Lu A.T., Quach A. (2018). An epigenetic biomarker of aging for lifespan and healthspan. Aging.

[10] Cohen A.A., Milot E., Yong J. (2013). A novel statistical approach shows evidence for multi-system physiological dysregulation during aging. Mech Ageing Dev.

[11] Fried L.P., Tangen C.M., Walston J. (2001). Frailty in older adults: evidence for a phenotype. J Gerontol A Biol Sci Med Sci.

[12] Malyutina S., Chervova O., Maximov V., Nikitenko T., Ryabikov A., Voevoda M. (2024). Blood-based epigenetic age acceleration and incident colorectal cancer risk: findings from a population-based case-control study. Int J Mol Sci.

[13] Morales Berstein F., McCartney D.L., Lu A.T. (2022). Assessing the causal role of epigenetic clocks in the development of multiple cancers: a Mendelian randomization study. eLife.

[14] Kim S.J., Kim B.J., Kang H. (2017). Measurement of biological age may help to assess the risk of colorectal adenoma in screening colonoscopy. World J Gastroenterol.

[15] Mak J.K.L., McMurran C.E., Kuja-Halkola R. (2023). Clinical biomarker-based biological aging and risk of cancer in the UK biobank. Br J Cancer.

[16] Dugué P.A., Bassett J.K., Wong E.M. (2021). Biological aging measures based on blood DNA methylation and risk of cancer: a prospective study. JNCI Cancer Spectr.

[17] Fiorito G., Caini S., Palli D. (2021). DNA methylation-based biomarkers of aging were slowed down in a two-year diet and physical activity intervention trial: the DAMA study. Aging Cell.

[18] Horvath S., Raj K. (2018). DNA methylation-based biomarkers and the epigenetic clock theory of ageing. Nat Rev Genet.

[19] Müller D., Győrffy B. (2022). DNA methylation-based diagnostic, prognostic, and predictive biomarkers in colorectal cancer. Biochim Biophys Acta Rev Cancer.

[20] Jung G., Hernández-Illán E., Moreira L., Balaguer F., Goel A. (2020). Epigenetics of colorectal cancer: biomarker and therapeutic potential. Nat Rev Gastroenterol Hepatol.

[21] Zhou X., Xiao Q., Jiang F. (2023). Dissecting the pathogenic effects of smoking and its hallmarks in blood DNA methylation on colorectal cancer risk. Br J Cancer.

[22] Sudlow C., Gallacher J., Allen N. (2015). UK biobank: an open access resource for identifying the causes of a wide range of complex diseases of middle and old age. PLoS Med.

[23] Codd V., Denniff M., Swinfield C. (2022). Measurement and initial characterization of leukocyte telomere length in 474,074 participants in UK biobank. Nature Aging.

[24] Gao X., Geng T., Jiang M. (2023). Accelerated biological aging and risk of depression and anxiety: evidence from 424,299 UK biobank participants. Nature Commun.

[25] Kwon D., Belsky D.W. (2021). A toolkit for quantification of biological age from blood chemistry and organ function test data: bioage. GeroScience.

[26] Hanlon P., Nicholl B.I., Jani B.D., Lee D., McQueenie R., Mair F.S. (2018). Frailty and pre-frailty in middle-aged and older adults and its association with multimorbidity and mortality: a prospective analysis of 493 737 UK biobank participants. Lancet Public Health.

[27] American cancer society cancer risk and prevention. https://www.cancer.org/cancer/risk-prevention/diet-physical-activity/alcohol-use-and-cancer.html.

[28] Tang L., Li D., Ma Y., Cui F., Wang J., Tian Y. (2023). The association between telomere length and non-alcoholic fatty liver disease: a prospective study. BMC Med.

[29] Qiu W., Chavarro J., Lazarus R., Rosner B., J M. (2025). powerSurvEpi: power and sample size calculation for survival analysis of epidemiological studies. R package version 015. https://CRAN.R-project.org/package=powerSurvEpi.

[30] van Buuren S., Groothuis-Oudshoorn K. (2011). Mice: multivariate imputation by chained equations in R. J Stat Software.

[31] Tidycmprsk T.F., DD. S (2024). Competing risks estimation. R package version 1.1.0. https://github.com/MSKCC-Epi-Bio/tidycmprsk.

[32] Gadd D.A., Hillary R.F., Kuncheva Z. (2024). Blood protein assessment of leading incident diseases and mortality in the UK biobank. Nature Aging.

[33] Imai K., Keele L., Tingley D. (2010). A general approach to causal mediation analysis. Psychol Methods.

[34] Heiss A. Generating inverse probability weights for both binary and continuous treatments.

[35] McCartney D.L., Min J.L., Richmond R.C. (2021). Genome-wide association studies identify 137 genetic loci for DNA methylation biomarkers of aging. Genome Biol.

[36] Marttila S., Kananen L., Häyrynen S. (2015). Ageing-associated changes in the human DNA methylome: genomic locations and effects on gene expression. BMC Genomics.

[37] Min J.L., Hemani G., Hannon E. (2021). Genomic and phenotypic insights from an atlas of genetic effects on DNA methylation. Nat Genet.

[38] Võsa U., Claringbould A., Westra H.J. (2021). Large-scale cis- and trans-eQTL analyses identify thousands of genetic loci and polygenic scores that regulate blood gene expression. Nature Genet.

[39] Fernandez-Rozadilla C., Timofeeva M., Chen Z. (2023). Deciphering colorectal cancer genetics through multi-omic analysis of 100,204 cases and 154,587 controls of European and East Asian ancestries. Nature Genet.

[40] Rashkin S.R., Graff R.E., Kachuri L. (2020). Pan-cancer study detects genetic risk variants and shared genetic basis in two large cohorts. Nat Commun.

[41] Hemani G., Zheng J., Elsworth B. (2018). The MR-Base platform supports systematic causal inference across the human phenome. eLife.

[42] Davies N.M., Holmes M.V., Davey Smith G. (2018). Reading Mendelian randomisation studies: a guide, glossary, and checklist for clinicians. BMJ.

[43] Rasooly D., Peloso G.M., Giambartolomei C. (2022). Bayesian genetic colocalization test of two traits using coloc. Curr Protoc.

[44] Shi X., Li M., Yao J., Li M.D., Yang Z. (2024). Alcohol drinking, DNA methylation and psychiatric disorders: a multi-omics Mendelian randomization study to investigate causal pathways. Addiction.

[45] Sun J., Zhao J., Zhou S. (2024). Systematic investigation of genetically determined plasma and urinary metabolites to discover potential interventional targets for colorectal cancer. J Natl Cancer Inst.

[46] Wu Y., Zeng J., Zhang F. (2018). Integrative analysis of omics summary data reveals putative mechanisms underlying complex traits. Nat Commun.

[47] Montégut L., López-Otín C., Kroemer G. (2024). Aging and cancer. Mol Cancer.

[48] Hägg S., Jylhävä J. (2020). Should we invest in biological age predictors to treat colorectal cancer in older adults?. Eur J Surg Oncol.

[49] Kotas M.E., Medzhitov R. (2015). Homeostasis, inflammation, and disease susceptibility. Cell.

[50] Lu A.T., Quach A., Wilson J.G. (2019). DNA methylation GrimAge strongly predicts lifespan and healthspan. Aging.

[51] Bian L., Ma Z., Fu X. (2024). Associations of combined phenotypic aging and genetic risk with incident cancer: a prospective cohort study. eLife.

[52] Dugué P.A., Bodelon C., Chung F.F. (2022). Methylation-based markers of aging and lifestyle-related factors and risk of breast cancer: a pooled analysis of four prospective studies. Breast Cancer Res.

[53] Liu Z., Leung D., Thrush K. (2020). Underlying features of epigenetic aging clocks in vivo and in vitro. Aging Cell.

[54] Harris K.M., Levitt B., Gaydosh L. (2024). Sociodemographic and lifestyle factors and epigenetic aging in US young adults: NIMHD social epigenomics program. JAMA Network Open.

[55] Maunakea A.K., Phankitnirundorn K., Peres R. (2024). Socioeconomic status, lifestyle, and DNA methylation age among racially and ethnically diverse adults: NIMHD social epigenomics program. JAMA Network Open.

[56] Li Y., Wang K., Jigeer G. (2024). Healthy lifestyle and the likelihood of becoming a Centenarian. JAMA Network Open.

[57] Tamayo M., Olivares M., Ruas-Madiedo P. (2024). How diet and lifestyle can fine-tune gut microbiomes for healthy aging. Annu Rev Food Sci Technol.

[58] Curnis F., Gasparri A., Sacchi A., Longhi R., Corti A. (2004). Coupling tumor necrosis factor-alpha with alphaV integrin ligands improves its antineoplastic activity. Cancer Res.

[59] Capri M., Salvioli S., Sevini F. (2006). The genetics of human longevity. Ann N Y Acad Sci.

[60] Napolioni V., Carpi F.M., Giannì P. (2011). Age- and gender-specific epistasis between ADA and TNF-α influences human life-expectancy. Cytokine.

[61] Huang X., Qin S., Liu Y., Tao L., Jiang H. (2019). Associations of tumor necrosis factor-α polymorphisms with the risk of colorectal cancer: a meta-analysis. Biosci Rep.

[62] Winnepenninckx B., Debacker K., Ramsay J. (2007). CGG-repeat expansion in the DIP2B gene is associated with the fragile site FRA12A on chromosome 12q13.1. Am J Hum Genet.

[63] Houlston R.S., Cheadle J., Dobbins S.E. (2010). Meta-analysis of three genome-wide association studies identifies susceptibility loci for colorectal cancer at 1q41, 3q26.2, 12q13.13 and 20q13.33. Nat Genet.

[64] Closa A., Cordero D., Sanz-Pamplona R. (2014). Identification of candidate susceptibility genes for colorectal cancer through eQTL analysis. Carcinogenesis.

[65] Huang C., Li H., Xu Y. (2023). BICC1 drives pancreatic cancer progression by inducing VEGF-independent angiogenesis. Signal Transduct Target Ther.

[66] Sun H., Li H., Guan Y. (2024). BICC1 drives pancreatic cancer stemness and chemoresistance by facilitating tryptophan metabolism. Sci Adv.

[67] Zhao R., Peng C., Song C. (2020). BICC1 as a novel prognostic biomarker in gastric cancer correlating with immune infiltrates. Int Immunopharmacol.

[68] Paolillo R., Boulanger M., Gâtel P. (2022). The NADPH oxidase NOX2 is a marker of adverse prognosis involved in chemoresistance of acute myeloid leukemias. Haematologica.

[69] Li L., Mao R., Yuan S. (2024). NCF4 attenuates colorectal cancer progression by modulating inflammasome activation and immune surveillance. Nat Commun.

[70] Yang S., Tang D., Zhao Y.C. (2021). Potentially functional variants of ERAP1, PSMF1 and NCF2 in the MHC-I-related pathway predict non-small cell lung cancer survival. Cancer Immunol Immunother.

[71] Prattichizzo F., Frigé C., Pellegrini V. (2024). Organ-specific biological clocks: ageotyping for personalized anti-aging medicine. Ageing Res Rev.

[72] Slieker R.C., Bos S.D., Goeman J.J. (2013). Identification and systematic annotation of tissue-specific differentially methylated regions using the illumina 450k array. Epigenetics Chromatin.

