# Supplementary material for: Accelerated biological aging and its hallmarks in DNA methylation drive the association between unhealthy lifestyles and the onset of colorectal cancer
Source: eBioMedicine. 2025 Nov 5;122:106005. doi: 10.1016/j.ebiom.2025.106005 (PMC12634281; doi:10.1016/j.ebiom.2025.106005)
Supplement: Supplementary Methods and Figures [file mmc1.docx]

**Supplementary Methods for**

**Accelerated biological aging and its hallmarks in DNA methylation drive the association between unhealthy lifestyles and the onset of colorectal cancer**

**Supplementary Methods**

***Assessment of biological aging***

In this cohort study, we employed multidimensional indicators including individual biomarker-based leukocyte telomere length (LTL) ^1^, clinical indicators and biomarkers-based PhenoAge acceleration ^2^, KDM-BA acceleration ^3^, and HD score ^4^, and clinical assessment-based FP ^5^ to quantify biological aging.

In UKBB, LTL was assessed by quantitative polymerase chain reaction technique, and the specific details have been reported elsewhere ^1^. Leukocyte DNA was obtained from participants’ peripheral blood samples with an automated process, and then amplified by qPCR method. Telomere length was quantified as a ratio of telomere repeats copy number to single gene copy number. The indicator was further loge-transformed and z-standardized, considering differences between laboratories in calibration samples and standard curves. PhenoAge was calculated using chronological age and 9 blood chemistries, including alkaline phosphatase, albumin, C-reactive protein, creatinine, glucose, mean cell volume, red cell distribution width, white blood cell count, and lymphocyte proportion, with aging-related mortality as the output variable in training ^2^. KDM-BA was calculated from systolic blood pressure, forced expiratory volume in one second, and 7 blood chemistry parameters (i.e., alkaline phosphatase, albumin, blood urea nitrogen, C-reactive protein, creatinine, glycated hemoglobin, and total cholesterol), using chronological age as the output variable in training ^3^. HD score utilizes the Mahalanobis distance ^4^ to assess how unusual an overall profile of biomarkers (i.e., albumin, alkaline phosphatase, C-reactive protein, total cholesterol, creatinine, glycated hemoglobin, systolic blood pressure, blood urea nitrogen, uric acid, lymphocyte percent, mean cell volume, white blood cell count) is for a given participant compared to a reference population. The NHANES III nonpregnant participants aged 20-30 years were used as reference samples for whom all user-selected biomarkers fall within the clinically normal range. It reflects the deviation of the person’s physiology from the relatively young and healthy reference sample, implying the degree of multi-system physiological dysregulation that is a key biological mechanism of aging ^6^. Following established practice for this measure, the natural logarithm was utilized to approximate normality. To quantify the deviation between biological age and chronological age, PhenoAge acceleration or KDM-BA acceleration was defined as the residual values computed by the regression of PhenoAge or KDM-BA relative to chronological ages at the time of biomarker measurement. To make effect sizes for the two measures of biological aging comparable, we standardized them to have a mean value of 0 and a standard deviation (SD) of 1 for analysis of a continuous dimension. Residuals were not estimated for HD score because it was not an age measure ^7^. The R package ‘BioAge’ ^8^ was employed to calculate these indices of biological aging. Details of used variables for the construction of biological aging are shown in **Supplementary Table 1**.

Frailty was measured using the FP ^5^, which was evaluated using 5 criteria (i.e., grip strength, exhaustion, weight loss, physical activity, walking speed) ^9^. Of them, weakness was measured by objectively measured handgrip strength, the others were assessed using a self-reported questionnaire (**Supplementary Table 2**). The FP score ranged from 0 to 5, with a higher score indicating more severe frailty. Participants were categorized into three groups (non-frail: FP score=0, pre-frail: FP score≥1 to ≤2, and frail: FP score≥3), following previous studies ^5,9^.

**References**

1. Codd V, Denniff M, Swinfield C, et al. Measurement and initial characterization of leukocyte telomere length in 474,074 participants in UK Biobank. *Nature aging.* 2022;2(2):170-179.

2. Levine ME, Lu AT, Quach A, et al. An epigenetic biomarker of aging for lifespan and healthspan. *Aging.* 2018;10(4):573-591.

3. Klemera P, Doubal S. A new approach to the concept and computation of biological age. *Mechanisms of ageing and development.* 2006;127(3):240-248.

4. Cohen AA, Milot E, Yong J, et al. A novel statistical approach shows evidence for multi-system physiological dysregulation during aging. *Mechanisms of ageing and development.* 2013;134(3-4):110-117.

5. Fried LP, Tangen CM, Walston J, et al. Frailty in older adults: evidence for a phenotype. *The journals of gerontology Series A, Biological sciences and medical sciences.* 2001;56(3):M146-156.

6. Li Q, Wang S, Milot E, et al. Homeostatic dysregulation proceeds in parallel in multiple physiological systems. *Aging cell.* 2015;14(6):1103-1112.

7. Mak JKL, McMurran CE, Kuja-Halkola R, et al. Clinical biomarker-based biological aging and risk of cancer in the UK Biobank. *British journal of cancer.* 2023;129(1):94-103.

8. Kwon D, Belsky DW. A toolkit for quantification of biological age from blood chemistry and organ function test data: BioAge. *GeroScience.* 2021;43(6):2795-2808.

9. Hanlon P, Nicholl BI, Jani BD, Lee D, McQueenie R, Mair FS. Frailty and pre-frailty in middle-aged and older adults and its association with multimorbidity and mortality: a prospective analysis of 493 737 UK Biobank participants. *The Lancet Public health.* 2018;3(7):e323-e332.

**Supplementary Figures**

**Supplementary Figure 1** Flowchart of the sample selection. CRC, colorectal cancer; EOCRC, early-onset CRC; LOCRC, late-onset CRC.

**Supplementary Figure 2** The correlation of the biological aging measures with chronological age based on (A) Pearson correlation and (B) Spearman correlation. The sample size was 322,640. LTL, leukocyte telomere length; HD, homeostatic dysregulation; KDM-BA, Klemera-Doubal method biological age.


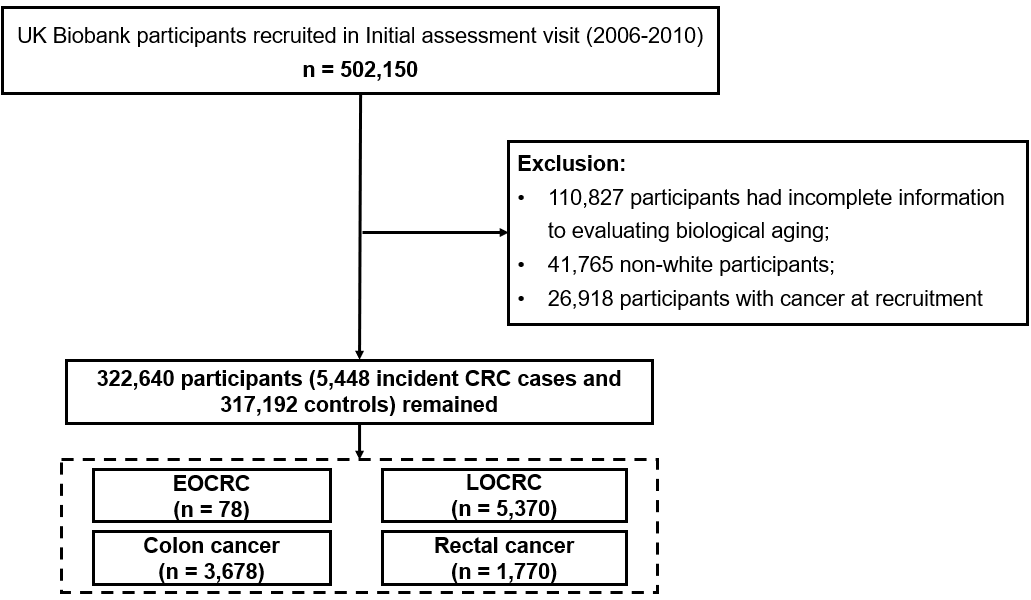


**Supplementary Figure 1** Flowchart of the sample selection. CRC, colorectal cancer; EOCRC, early-onset CRC; LOCRC, late-onset CRC.


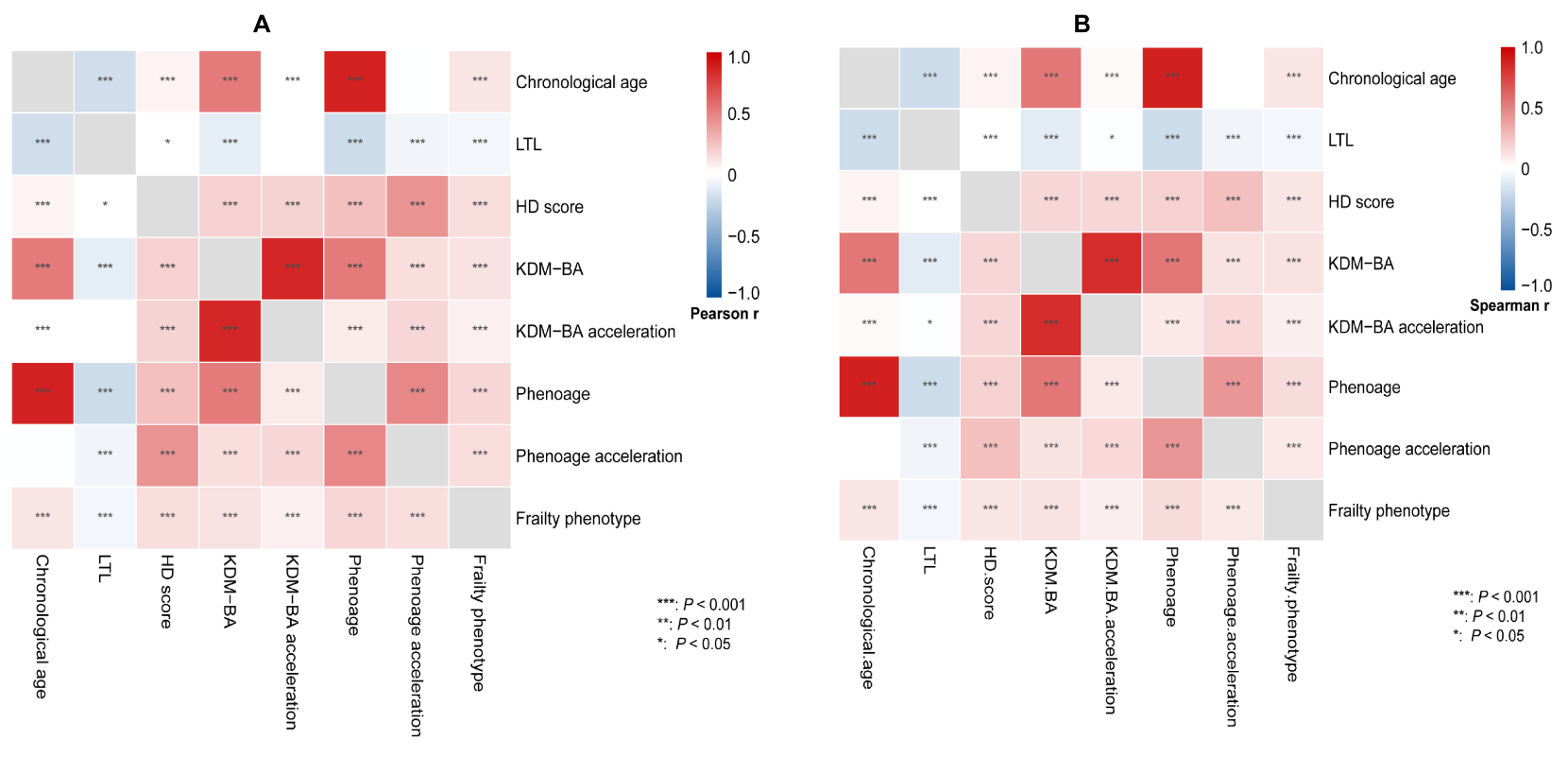


**Supplementary Figure 2** The correlation of the biological aging measures with chronological age based on (A) Pearson correlation and (B) Spearman correlation. The sample size was 322,640. LTL, leukocyte telomere length; HD, homeostatic dysregulation; KDM-BA, Klemera-Doubal method biological age.
